# The Prevalence of Pediculosis in Southern Iran From 2016 to 2021

**DOI:** 10.1155/jotm/5098369

**Published:** 2025-12-05

**Authors:** Aboozar Soltani, Kourosh Azizi, Ali Poushpas, Rahil Hamedpour, Mohammad Mahdi Parvizi, Seyed Arshiahossein Fazelzadeh Haghighi, Negin Fazelzadeh Haghighi

**Affiliations:** ^1^ Department of Medical Entomology and Vector Control, School of Health, Research Center for Health Sciences, Institute of Health, Shiraz University of Medical Sciences, Shiraz, Iran, sums.ac.ir; ^2^ Molecular Dermatology Research Center, Shiraz University of Medical Sciences, Shiraz, Iran, sums.ac.ir; ^3^ Department of Dermatology, Shiraz University of Medical Sciences, Shiraz, Iran, sums.ac.ir

**Keywords:** ectoparasitic infestations, epidemiologic studies, head, Iran, lice infestations, *Pediculus*

## Abstract

**Introduction:**

One important measure of community and personal hygiene is the prevalence of lice. Healthcare providers and strategists must analyze this illness across several groups if they are to create sensible plans and services for its decrease. The objective of this study was to find the prevalence of head lice in Iranian endemic regions.

**Methods:**

In this retrospective cross‐sectional study, all patients referred to healthcare centers affiliated with Shiraz University of Medical Sciences from 2016 to 2021 who were diagnosed with head lice were included. Those confirmed with head lice were isolated and examined by a physician of the healthcare center. Patient information, including city, diagnosis date, type of disease, age, sex, nationality, and urban or rural status, was recorded. Data analysis was performed using descriptive methods in SPSS Version 24 and STATA 14.2.

**Results:**

The results showed a total of 153,550 cases of lice infection (average annual prevalence 0.52% [95% CI: 0.52% to 0.53%]), with a declining trend from 20,951 infections to 5912 during the study period. The highest infection rate (49.3%) was observed in the 6–12 age range; most infected patients, 93.2%, were female. Furthermore, Qirokarzin, Zarrin Dasht, Mohr, and Rostam displayed the highest infestation levels, accounting for 55.4% of infections among the urban population.

**Conclusion:**

Overall, the present study revealed a low prevalence of head lice in Fars Province, southern Iran; most of the individuals with head lice were elementary school‐aged girls. Therefore, it is important to focus on interventions and policy‐making with the aim of preventing infestation and the spread of disease among this population. Moreover, the general drop in yearly infestation shows how well Shiraz University of Medical Sciences’ policies regulate the disease.

## 1. Introduction

Lice are parasites of mammals and birds [1, 2]. Head louse (*Pediculus humanus capitis*), body louse (*Pediculus humanus corporis*), pubic louse (*Pthirus pubis*), and animal lice (various species such as *Trichodectes canis* and *Felicola subrostratus*) are hematophagous ectoparasites that are responsible for human pediculosis [3–5].

Head lice live in human hair, feeding on blood several times per day and attaching their eggs (nits) to the hair shafts, while body lice reside primarily within the seams of clothing, laying eggs there, and feeding less frequently. Body lice evolved from head lice when people started dressing up. Genetically, the head lice are the more diverse group with six family lines (Clades A through F), while the body lice adhere to just two (A and D), indicating that they are the new kids on the block. Body lice are typically associated with poor hygiene and overcrowded living conditions, such as poor or crowded living conditions, and can spread serious diseases such as epidemic typhus, trench fever, and relapsing fever, thereby representing a major public health concern [6–8].

The head louse is smaller, darker, and has a more resistant covering than the body louse, which shows minor differences. In severe cases, head lice may be found on the body, but body lice are never found on the head [3, 9]. Head lice infestations primarily occur in children aged 3–10 and their families, affecting about 3% of schoolchildren in the United States. Individuals of African descent are seldom affected due to differences in hair texture. Head lice spread through direct head‐to‐head contact with an infected person [10].

The three principal phases in the life cycle of lice are egg, nymph, and adult. When the eggs (or nits) hatch, which will be 6–9 days depending on conditions such as temperature, the lice are in the nymph stage. The lice further divide their life cycle into three distinct instars, or developmental stages. Each nymphal phase takes a few days (typically 2–5 days per instar) during which the nymphal molt of head lice shed their exoskeleton as they grow larger. To this end, the life cycle of lice contains three instars: first‐instar nymphs (immediately after hatching), second‐instar nymphs (after first molting), and third‐instar nymphs (after second molting). After the third molt, they are fully grown adults that can feed and reproduce. Thus, the nymph stage takes around 9–12 days prior to adulthood [11–13].

Though they cannot pierce the skin, lice feed on blood by piercing their needle‐like mouthparts, which irritates and causes itching from their saliva [14]. Usually, developing 3–4 weeks following the first infestation, scalp itching is the main sign of an infestation. Excessive scratching commonly causes excoriations that are prone to secondary bacterial infection [15]. The diagnosis is careful combing of the scalp with a fine‐toothed comb, looking for live lice in the comb teeth following each pass [16]. In the United States, almost 14 million people, mostly children, are treated for head lice annually; yet, only a small percentage display obvious symptoms of an infestation [17]. Significant lice infestations have also been reported globally, particularly in Denmark, Sweden, Britain, France, and Australia. [18]. The National Health Service in Britain, along with various health agencies in the United States, indicates that head lice tend to prefer clean hair. This preference arises because clean hair provides a more suitable surface for them to attach their eggs, which can adhere more easily to the strands [19].

Head lice remain inadequately controlled and are an epidemiological public health problem due to several reasons; therefore, the frequency of lice is an important indicator of personal and community hygiene [20, 21]. Underreporting and delayed treatment due to social stigma associated with lice, lack of uniform school screening policy, and public unawareness hinder successful prevention. In addition, lice are developing resistance to standard treatments, and improper use of pediculicides reduces their efficiency. Reinfestation is common, particularly when the individuals in close contact are not treated simultaneously. Head lice are not a fatal illness but can be easily spread in group settings such as schools, resulting in discomfort, absenteeism, and social stigmatization of children and emotional and financial strain on families [20, 22–24].

In recent years, evidence has revealed that different types of lice are still a common issue for both people and animals in Iran. In this regard, head lice (*Pediculus humanus capitis*) and body lice (*Pediculus humanus corporis*) are most frequently encountered in humans, with school‐aged children being the most affected group. Provinces such as Tehran, Khuzestan, and Sistan and Baluchestan have reported higher rates of these infestations. On the animal side, lice such as *Haematopinus*, *Bovicola*, and *Linognathus* species are frequently found on livestock, particularly sheep, goats, and cattle, mostly in rural and pastoral communities. These findings highlight how lice remain a public and veterinary health concern in many parts of Iran [25–29].

It is crucial to evaluate the frequency of head lice infestations in diverse demographic groups. Analyzing this condition using several demographic characteristics helps healthcare professionals to develop important insights in creating more successful policies and services meant to address this ongoing problem. Such assessments help public health campaigns to be more suited to the particular needs of groups afflicted with head lice [30, 31]. Thus, this study aimed to investigate the prevalence of head lice, particularly in areas covered by Shiraz University of Medical Sciences in southern Iran, during the period from 2016 to 2021, using data recorded by the Vice‐Chancellor for Health in Shiraz University of Medical Sciences.

## 2. Methods

### 2.1. Study Design and Participants

This is a retrospective cross‐sectional study conducted according to the data recorded on the online registry system for infectious diseases affiliated with the Vice‐Chancellor of Shiraz University of Medical Sciences. The study population comprised all patients with head lice who were referred to medical centers affiliated with Shiraz University of Medical Sciences in Fars Province, Iran, from 2016 to 2021. Each patient was assessed based on the following data:

1. City name.
2. Year of diagnosis.
3. Month of diagnosis.
4. Type of infestation (body, head, or pubic lice).
5. Age group (under 6 years, 6–12 years, 12–18 years, and over 18 years).
6. Gender (male or female)
7. Nationality (Iranian or non‐Iranian).
8. Location (urban or rural).

### 2.2. Inclusive and Exclusion Criteria

All the patients whose head louse was confirmed clinically by the physicians of the health centers affiliated with Shiraz University of Medical Sciences, whose information was recorded in the web‐based panel of the chancellery of health in Shiraz University of Medical Sciences, were included in the study. If the diagnoses of the registered patients were not recorded in this system, the case was removed from data analyses.

### 2.3. Ethical Considerations

The Ethics Committee of Shiraz University of Medical Sciences approved the protocol of this study (IR.SUMS.MED.REC.1402.099). When their head lice were confirmed and their data were recorded in a related web‐based panel, the patients were given a written informed consent form to use their data in any research project anonymously. For patients under 7, written informed consent was obtained from their parents; for those aged 7–18, consent was obtained from both the child and their parents. Furthermore, we received the anonymized data file from the Vice‐Chancellor of Health in Shiraz University of Medical Sciences, Shiraz, Iran.

### 2.4. Statistical Analyses

We used the latest census data for Fars Province to estimate the prevalence of the disease during the study period [32]. So, the population estimates were used as denominators to calculate the prevalence of head lice. Descriptive statistics, including frequencies and percentages for qualitative variables, were used to summarize the data. In addition, the data were examined for missing values, outliers, or inconsistencies. Missing data on demographic characteristics were reported accordingly. Data analysis was conducted using SPSS Version 24. Moreover, for the calculation of the 95% confidence interval (CI), we used the “cii proportion” of STATA software Version 14.2.

## 3. Results

**Figure 1:**
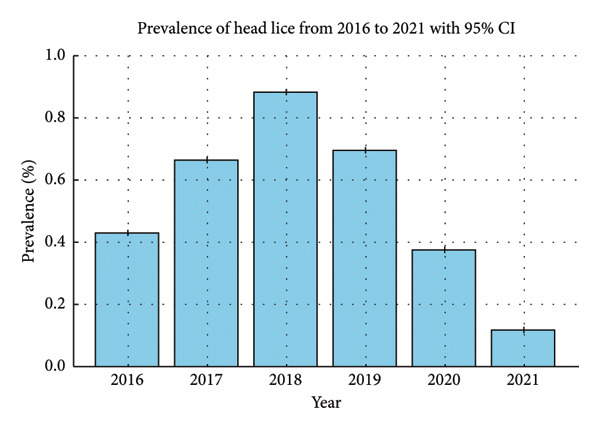
The prevalence of head lice from 2016 to 2021. This bar chart illustrates the prevalence of head lice infestation from 2016 to 2021, along with the 95% confidence interval (95% CI). It can be observed that the highest prevalence occurred in 2018 (0.88%), while the lowest was recorded in 2021 (0.12%).

The study identified 153,550 cases of head lice infestation from 2016 to 2021, resulting in an average annual prevalence of 0.52% (95% CI: 0.52%–0.53%) based on the latest census data for Fars Province, which reported that 4,851,274 individuals lived in Fars Province, Iran. [32]. As shown in Table 1 and Figure 1, the prevalence of head lice increased from 2016 to 2019 but started to fall thereafter, reaching its lowest point in 2021 with 5912 cases.

**Table 1:** The prevalence of head lice from 2016 to 2021.

		Frequency	Prevalence (95% CI)^a^
Year	2016	20951	0.43% (0.42 to 0.44)
	2017	32237	0.66% (0.65 to 0.67)
	2018	42851	0.88% (0.87 to 0.89)
	2019	33370	0.69% (0.68 to 0.70)
	2020	18229	0.38% (0.37 to 0.38)
	2021	5912	0.12% (0.11 to 0.13)

^a^CI, confidence interval.

**Figure 2:**
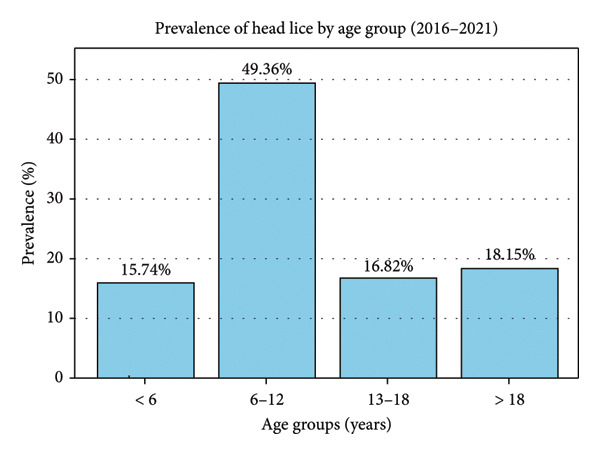
Head lice prevalence across age groups among patients registered at Shiraz University Health Centers (2016–2021). This bar chart shows the prevalence of head lice infestation across different age groups between 2016 and 2021. The highest prevalence is observed in the 6–12‐year age group (49.36%), while the lowest prevalence is seen among children under 6 years old.

Moreover, Table 2 and Figure 2 demonstrate that 143,115 patients (93.2%) were female, while 10,435 (6.8%) were male, indicating a higher prevalence among females. Of the individuals with infestations, 75,787 (49.3%) were aged 6–12 years, 27,867 (18.1%) over 18 years, 25,820 (16.8%) 13 to 18 years, and 24,174 (15.7%) were under 6 years, indicating that the highest number of cases occurred in the 6–12 age group. Among those with head lice, 85,088 individuals (55.4%) were from urban areas, while 68,462 (44.6%) were from rural areas, showing a predominance in urban settings. The cities of Qirokarzin (3606.82 per 100,000 population), Zarrin Dasht (3342.49 per 100,000), Mohr (3241.70 per 100,000), and Rostam (2323.18 per 100,000) had the highest prevalence, whereas Eqlid (50.66 per 100,000) and Abadeh (113.56 per 100,000) recorded the lowest rates.

**Table 2:** Prevalence of head lice (2016–2021) by gender, age, and residence of patients registered at Shiraz University of Medical Sciences Healthcare Centers.

Variable		Frequency	Percent
Gender	Female	143115	93.2
	Male	10435	6.8

Age groups (Year)	< 6	24174	15.74
	6 to 12	75785	49.36
	13–18	25820	16.82
	> 18	27867	18.15
	Undetermined	96	—

Location	Urban	85088	55.41
	Rural	68462	44.59

## 4. Discussion

The current study found a prevalence of about 0.52% for head lice infestation in Fars Province from 2016 to 2021. The other study conducted by Ziaoddini et al. reported the prevalence of 0.34%–1.53% among the students in Fars Province, Iran [33]. The mean global prevalence of head lice was estimated at 0.25% among preschool and primary school students and the community [34]. The study conducted by Nasirian et al. in 2024 showed that the prevalence of this disease had increased in half of the provinces in Iran over the past 2 decades [35]. Previous studies revealed that several factors, such as female gender and the summer season, increased the risk of infection, while autumn and winter played a protective role [34, 36].

While there are no other new studies specific to Fars Province, the study conducted by Jalalyer in 1967 in a village near Shiraz, Fars, Iran, revealed that more than 30% of the patients were infected with *Pediculus humanus capitis* and that more than 75% of them were girls and women [37]. However, Habibi et al. reported that only 1.17% of the population in Mazandaran Province, the northernmost part of Iran, was affected [38]. The declining frequency observed in this study may be attributed to increased public awareness and improvements in hygiene standards over time. Although the overall trend of infestation decreased throughout the study period, a notable surge was recorded between 2016 and 2019. This aligns with Ziaaldini et al.’s findings, which showed a rising trend in head lice infestation among urban students from 2014 to 2018, mirroring the upward trend observed in this study until 2019. The increase in diagnosis accuracy and reporting was mentioned as a reason for the upward trend in that study [33].

Increased public awareness and the execution of instructional programs in schools help to explain the general decline in head lice infection [30, 38–41]. Furthermore, it is possible that the COVID‐19 epidemic, which led to quarantine policies in late 2019, contributed to this tendency; as noted by Galassi et al., head lice frequency dropped significantly during the quarantine when compared to earlier years. Furthermore, in households with two or fewer children, infestations were more under control than in those with three or more [16].

In the present study, women comprised most of the head lice patients. Likewise, Amelia et al. found that women had more infestation [42]. With 93% of those impacted identified as females, Amirkhani et al. found that this closely matched the gender pattern noted in this study [43]. There are various reasons for the greater head lice infection seen in girls than in boys. Their hair thickness and size are one important consideration since they create a more suitable habitat for lice to flourish. In addition, among females with abundant or long hair, the frequency of hair washing and routine hygiene practices may be reduced, as maintaining such hair can be more time‐consuming and challenging. Close physical proximity to peers, particularly in school settings, further facilitates the transmission of lice among young individuals [44].

The current study results, in the same line with other studies which claimed this age range as the most affected, found the highest prevalence of head lice infestation in the 6–12 age group [45]. Castro et al. also observed the greatest prevalence among youngsters aged 10–12 [46]. In addition, 55.4% of the cases in the current study were from urban populations, which contrasts with Lashkari et al., who found that peri‐urban areas had twice the prevalence of urban areas [47]. Furthermore, Habibi et al. indicated that rural areas had a significantly higher prevalence as well [38].

In cities including Qirokarzin, Zarrin Dasht, Mohr, and Rostam, head lice infection rates point to a major public health issue. The lowest infestation rates shown by the cities of Eqlid and Abadeh point to a possible difference in environmental or other determinant variables. The differences in head lice infestation rates among these numerous cities can be associated with several underlying elements, including the degrees of education regarding lice prevention, the general hygienic standards maintained by the population, and the availability of employment possibilities. These factors can greatly influence how communities handle head lice epidemics, which will clearly vary the infestation rates among the cities [41].

Furthermore, evidence shows that population density and climate conditions are major determinants of the frequency of head lice infestations. For example, Kassiri’s research revealed that the prevalence of head lice in Khuzestan Province increased significantly in densely populated areas with warm and humid environmental conditions [48]. Other areas, notably Egypt, where the interaction of warm, humid temperatures and dense populations has produced similar results regarding head lice infestations, have also experienced this trend of increasing frequency due to similar environmental and demographic elements [49].

There were some limitations in this study. First, the web‐based panel did not record some of the principal sociodemographic characteristics of the patients, including their race, educational status, marital status, economic status, and living address details. Therefore, we did not have more patient details for sensitivity analyses. Second, the panel failed to record the patients’ disease stages. In addition, it is possible that some patients with head lice were referred to private dermatology clinics or general physicians’ clinics, so their information was not registered in the panel, and some of the patients tended to self‐treat, so these patients were not included in our data analyses. On the other hand, it is important to note a limitation of passive screening for infested students, as only individuals who are already infested are typically referred to health centers. Therefore, the potential underestimation of this method compared to active screening should be acknowledged. These limitations may affect the overall findings and conclusions of our study, as the absence of comprehensive patient data could skew the results. Future research should aim to include a broader range of patient information and consider referrals to other healthcare providers to ensure a more complete understanding of head lice management. The authors strongly recommended that these issues should be considered in further studies.

## 5. Conclusion

This study indicated a head lice prevalence of 0.52% in Fars Province, with more than 93% of cases found among 6–12‐year‐old girls. These data underscore that primary school girls represent the most population vulnerable to infestation. Enhancing focused prevention and educational initiatives for parents, educators, and school personnel may significantly contribute to a decrease in transmission. Improving community knowledge and sustaining coordinated public health activities are equally vital to preserving the advancements made in head lice management and preventing future outbreaks.

## Disclosure

The funders had no role in the study design, data collection, analysis, decision to publish, or manuscript writing.

## Conflicts of Interest

The authors declare no conflicts of interest.

## Author Contributions

The study was designed and coordinated by Aboozar Soltani. Kourosh Azizi participated in drafting the manuscript and reviewing of the literature. Ali Poushpas collected and analyzed the data and participated in editing the manuscript. Rahil Hamedpour participated in editing the manuscript and literature review. Mohammad Mahdi Parvizi participated in the study design, edited the manuscript, and analyzed the data. Seyed Arshiahossein Fazelzadeh Haghighi participated in data collection and literature review. Negin Fazelzadeh Haghighi participated in designing the study, writing the manuscript, and reviewing the literature.

## Funding

This study was financially supported by the Shiraz University of Medical Sciences (Grant no. 27716).

## Data Availability

The data that support the findings of this study are available from the corresponding author upon reasonable request.
